# The Role of Cardiac Magnetic Resonance in Characterizing Atrial Cardiomyopathy and Guiding Substrate Ablation in Atrial Fibrillation: A Narrative Review

**DOI:** 10.3390/jcdd12040114

**Published:** 2025-03-25

**Authors:** Jean Pierre Jabbour, Marta Palombi, Michela Bonanni, Andrea Matteucci, Luca Arcari, Nicola Pierucci, Vincenzo Mirco La Fazia, Carlo Lavalle, Marco Valerio Mariani

**Affiliations:** 1 Department of Cardiovascular, Respiratory, Nephrological, Anesthesiological and Geriatric Sciences, “Sapienza” University of Rome, 00185 Rome, Italy; 2 Department of Experimental Medicine, Tor Vergata University, 00133 Rome, Italy; 3 Clinical and Rehabilitation Cardiology Division, San Filippo Neri Hospital, 00135 Rome, Italy; 4 Cardiology Unit, Madre Giuseppina Vannini Hospital, Via di Acqua Bullicante 4, 00177 Rome, Italy; 5 Department of Internal Clinical, Anesthesiological and Cardiovascular Sciences, “Sapienza” University of Rome, 00185 Rome, Italy

**Keywords:** cardiac magnetic resonance, atrial cardiomyopathy, atrial fibrillation, atrial fibrillation ablation

## Abstract

Cardiac magnetic resonance imaging (MRI) is increasingly recognized as a promising tool for tissue characterization in atrial fibrillation (AF), providing detailed insights into anatomy, fibrosis, and scarring. While MRI cannot directly guide ablation lesions, its ability to identify arrhythmogenic substrates could improve patient stratification and procedural planning. Despite these theoretical advantages, the clinical utility of MRI in guiding substrate-based ablation strategies remains a matter of debate. Methods: Our review evaluates the current evidence supporting the integration of MRI into the workflow of AF ablation. Specifically, we examine findings from randomized trials and prospective studies that have investigated the predictive value of MRI-derived fibrosis quantification for procedural outcomes and arrhythmia recurrence. We aim to assess whether MRI can enhance the personalization of ablation strategies and predict treatment success. Challenges such as variability in imaging protocols, lack of standardization in fibrosis quantification, and limited large-scale validation are also addressed. This review provides a comprehensive overview of the current status and potential of MRI in the evolving field of AF ablation.

## 1. Introduction

Atrial fibrillation (AF) is the most commonly diagnosed arrhythmia in clinical practice, particularly among the elderly [1]. It represents an increasingly significant healthcare issue worldwide as it is associated with substantial morbidity and mortality, primarily due to heart failure (HF) and thromboembolic events [2]. Current evidence favors a rhythm control strategy over rate control, demonstrating benefits in symptom relief and quality of life (QoL) improvement. In this context, several studies have shown that AF catheter ablation is more effective than antiarrhythmic drug (AAD) therapy in maintaining sinus rhythm and alleviating symptoms, whether used as a first-line treatment or after AAD failure or intolerance [3,4,5,6,7]. Historically, AF has been regarded as a rhythm disturbance, resulting in uncoordinated atrial activation and the loss of effective atrial contraction. More recently, the concept of atrial cardiomyopathy (AtCM) has emerged as a possible substrate for AF [8]. The individuation of a pathological substrate is crucial as it affects the prognosis of patients with AF, regardless of the chosen treatment strategy or the outcome of catheter ablation. Patients with extensive atrial disease (i.e., atrial fibrosis) have worse outcomes after catheter ablation and tend to exhibit increased mortality and stroke risk, even in the absence of AF recurrence [9]. Cardiac magnetic resonance (CMR), widely implemented in the study of cardiomyopathies due to the possibility of performing tissue characterization, has been adopted to assess AtCM and identify atrial fibrosis. In this review, we aim to analyze the role of CMR in this context and its potential in guiding AF ablation procedures.

## 2. Methods

We conducted a narrative review following a transparent selection process. We performed a literature search in the PubMed, Scopus, and Web of Science databases, covering publications from January 2010 to December 2024. The search strategy included combinations of the following keywords: “atrial fibrillation”, “cardiac magnetic resonance”, “atrial cardiomyopathy”, “fibrosis”, “substrate ablation”, and “left atrium”. We included original articles, randomized controlled trials, prospective cohort studies, meta-analyses, and relevant review articles written in English. Studies were selected based on their relevance to the role of CMR in characterizing atrial cardiomyopathy and its potential application in guiding substrate-based ablation strategies. Case reports, editorials, and studies without a direct focus on AF or MRI-guided ablation were excluded.

## 3. AtCM and AF: Which Came First?

A recent consensus document of the European Heart Rhythm Association (EHRA), the Heart Rhythm Society (HRS), the Asian Pacific Heart Rhythm Society (APHRS), and the Latin American Heart Rhythm Society (LAHRS) defined AtCM as “any complex of structural, architectural, contractile or electrophysiological changes affecting the atria, with the potential to produce clinically relevant manifestations” [8]. This broad definition underlines the multifactorial origin of AtCM as its potential clinical manifestations include atrial arrhythmias (most commonly AF), cardioembolic events, HF events due to atrial failure, and atrial enlargement leading to valvular regurgitation. In the previous consensus document on AtCM published in 2016 [10], the working group proposed a histological classification for AtCM consisting of four different categories. The first category is characterized by cardiomyocyte dysfunction associated with genetic abnormalities, primarily in cardiomyopathy-related genes, especially in the subset of young patients with early-onset AF [11,12,13]. The second category refers to principally fibrotic changes, which can be observed in patients with known cardiovascular disease and those displaying advanced fibrotic disease despite a low-risk profile (e.g., young patients with few or no cardiovascular risk factors). The third category reflects a mixed dysfunction of both cardiomyocytes and fibroblasts, illustrating the complex interplay between clinical findings (such as left atrial (LA) enlargement, LA volume, and pressure overload) and cellular processes (including apoptosis, fibrosis, and electrical abnormalities). Lastly, the fourth category includes primarily non-collagen infiltration (with or without cardiomyocyte changes), as seen in deposition disease (e.g., cardiac amyloidosis, iron overload cardiomyopathy). This classification aims to simplify the understanding of the etiopathogenesis of the primary atrial disease; however, it is not broadly accepted due to the lack of routine execution of myocardial biopsies in these patients.

As AtCM exhibits a multifaceted pathophysiology, various additional factors contribute to its development. The role of inflammation has been well established, particularly through the overexpression of inflammatory signaling pathways [14]. Moreover, genetic and epigenetic regulation of innate immunity is thought to play a crucial role in modulating susceptibility to AF by influencing inflammatory responses [15]. In addition, factors such as aging, oxidative stress, atrial adipose tissue, and atrial fibro-fatty infiltration have been implicated in the development of atrial fibrosis, electrical remodeling, and a prothrombotic state, all of which contribute to an increased risk of AF [8,16,17,18,19,20]. It is important to distinguish AtCM from AF, as AF can be a consequence of AtCM; while AtCM frequently leads to AF, AF can also exacerbate AtCM. The mechanisms outlined earlier interact in a way that creates a vicious cycle, driving the progression of AtCM and the development or worsening of AF. In that way, AF tends to perpetuate itself, as depicted by the expression “AF begets AF” [21]. As a form of cardiomyopathy, AtCM progresses through distinct stages in its natural history. The initial stage is a mild subclinical phenotype, detectable only through electrophysiological measurements and/or imaging techniques, without manifest arrhythmias or LA dysfunction; the intermediate stage is characterized by significant structural abnormalities, impaired atrial function, elevated HF biomarkers, and/or clinical AF. The advanced stage (i.e., “atrial failure”) involves severe structural changes, pronounced impairment of atrial function, and/or persistent or permanent AF [8]. While AtCM progresses in severity, the presence and role of atrial fibrosis become increasingly evident, as it is a key pathophysiological component causally linked to the disease.

## 4. Atrial CMR Imaging for LA Fibrosis Evaluation: A New Frontier

**Figure 1:**
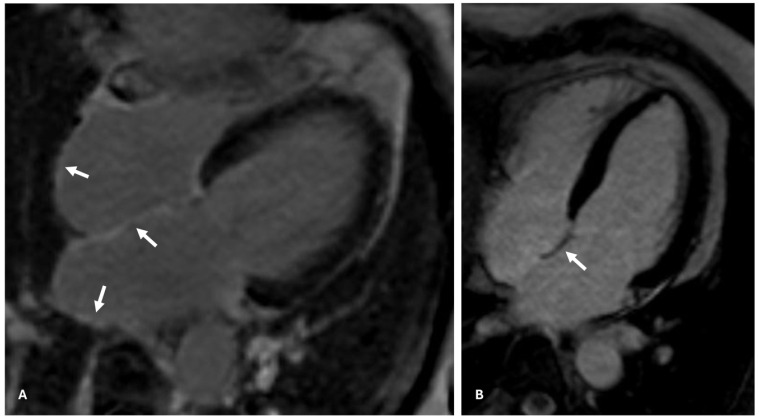
Delayed enhancement cardiac magnetic resonance imaging, long-axis 4-chamber view, in a patient with (arrows in (**A**)) and without (arrow in (**B**)) atrial fibrosis.

**Figure 2:**
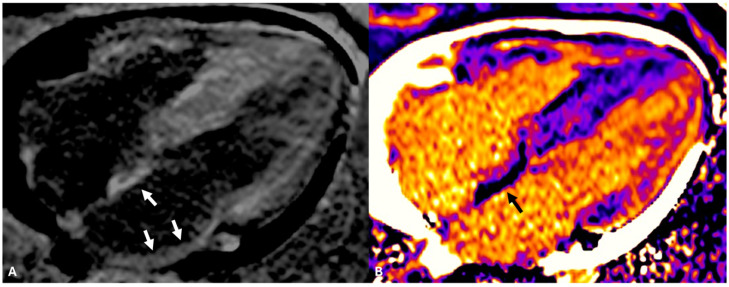
Delayed enhancement cardiac magnetic resonance imaging, long-axis 4-chamber view, in a patient affected by cardiac amyloidosis with extensive atrial fibrosis (arrows in (**A**)). The same view acquired with T1 post-contrast imaging is depicted in (**B**): the arrow highlights the low signal areas corresponding to the interatrial septum; however, less thickened regions of the left atrium wall are less well imaged due to a lower spatial resolution.

In AtCM, the development of myocardial fibrosis is a key factor driving cardiac remodeling. The significance of atrial fibrosis is not completely clear since not all tissue fibrosis is identical and two principal types can be identified: in “reactive” (interstitial) fibrosis, the proliferation and differentiation of fibroblasts, along with the accumulation of extracellular matrix (ECM) material in the perivascular space and perimysium, do not seem to significantly alter the muscular architecture or the tissue’s conduction properties, at least when thin interstitial strands are present; indeed, “replacement” (reparative) fibrosis, by replacing dead cardiomyocytes with ECM tissue and fibroblasts, may be much more disruptive to electric conduction and more difficult to reverse than reactive fibrosis [22], as it leads to localized regions of slow conduction, increases conduction heterogeneity, and creates a substrate for arrhythmias [23]. Postmortem histological studies have demonstrated a correlation between the degree of fibrosis and AF [24]. Furthermore, several studies have shown that the more advanced these abnormalities are, the lower the efficacy of AF ablation [25,26,27]. AtCM can be studied using imaging techniques; among these, CMR has the unique capability of performing tissue characterization and, therefore, assessing atrial fibrosis using three-dimensional (3D) late gadolinium enhancement (LGE)-CMR imaging. LGE may result from conditions that substantially increase the interstitial space or slow down gadolinium clearance: the LGE technique can delineate areas of fibrotic tissue by exploiting differences in gadolinium washout kinetics between normal and diseased myocardium. In LGE-CMR imaging, LA fibrosis is visualized as areas of high signal intensity on T1-weighted sequences due to gadolinium contrast accumulation [28] (Figure 1 and Figure 2). Supplementary Table S1 summarizes the atrial CMR imaging acquisition protocol adopted in the most important studies evaluating MRI-guided AF ablation.

Interestingly, AtCM has been proposed as a potential substrate for certain types of cryptogenic stroke [29]; a study found that the burden of LA LGE detected by CMR interacted with the risk of ischemic stroke, even in the absence of AF detected by ECG or a 24 h Holter ECG monitor [30]. This observation suggests that the diagnosis of AtCM through CMR imaging has prognostic value in these patients, even in the absence of documented clinical AF.

## 5. The Role of CMR in Planning AF Ablation

Pulmonary veins (PV) anatomy visualization is a key element in planning the AF ablation procedure; their number, location, and diameters are important information in the pre-procedural setting. CMR with angiographic sequences can visualize the anatomy of the PV, accurately measure their size and ostia, and detect anatomical variations, such as accessory PV or a common ostium, as well as abnormalities like anomalous PV return. In addition to this, the measurement of LA size is essential for risk stratification in patients with AF: LA enlargement has been identified as an independent risk factor and a predictor of both stroke and mortality in individuals diagnosed with AF [31]. While transthoracic echocardiography is the most widely adopted method in routine clinical practice for assessing LA size by deriving atrial volumes from two-dimensional (2D) measures, CMR is regarded as the gold standard for evaluating cardiac volumes, including LA volumes. The advantages of CMR stem from its technical capabilities; indeed, steady-state free precession (SSFP) techniques and angiographic 3D acquisitions do not rely on geometric assumptions, yielding superior accuracy [32].

Ablation procedures for AF are typically performed using a combination of fluoroscopic and electro-anatomical imaging techniques. Even with experienced operators, these procedures expose patients to a consistent radiation dose [33]. In the last few decades, the fusion of electro-anatomical imaging with pre-procedural CMR images has become a valuable approach in conducting ablation procedures, assisting electrophysiologists in reducing both procedural time and radiation exposure. This innovative integration also aids in the prevention of complications, such as esophageal thermal injury (ETI), which can have serious consequences. The integration of advanced cardiac imaging into electro-anatomical mapping offers detailed insights into patient anatomy, leading to more effective pulmonary vein isolation (PVI) and increased procedural safety [34].

## 6. Substrate AF Ablation Using CMR-Guided Approach

AF remains one of the most prevalent and challenging arrhythmias to manage; in a non-negligible percentage of cases, AF represents the arrhythmic manifestation of AtCM. The extent of the diseased substrate helps explain the presence of more treatment-resistant AF forms, especially in young patients with normal LA size on echocardiography; conversely, paroxysmal AF can also occur in patients with significant comorbidities and underlying heart disease, even in the presence of a more advanced structural condition [35]. AtCM plays a key role in both the initiation and maintenance of AF, while persistent or long-standing AF, in turn, accelerates the progression of AtCM. Restoring sinus rhythm through catheter ablation is the most effective strategy to disrupt this vicious cycle and prevent the further advancement of AtCM [8]. In recent years, the integration of CMR imaging into substrate-guided AF ablation has gained considerable attention due to its ability to non-invasively visualize atrial fibrosis, thereby enabling tailored ablation strategies that target not only the triggers but also the underlying substrate of the arrhythmia [25]. The ability to study atrial fibrosis by CMR is crucial because fibrotic regions in the atria have been correlated with heterogeneous conduction properties and the formation of re-entrant circuits that facilitate the perpetuation of AF [26]. Despite the solid and validated role of delayed-enhancement (DE)-CMR in characterizing substrate ablation in AF patients, conflicting outcomes have emerged from clinical studies aiming to evaluate its role in improving AF ablation outcomes. Table 1 shows the most important studies assessing the added value of DE-CMR in the ablation strategy of AF. Studies demonstrating positive results with DE-CMR-guided substrate AF ablation were observational. In particular, Quinto et al. [36] showed that the use of DE-CMR to guide PVI and substrate ablation in repeated procedures was associated with a shorter procedural time and lower risk of recurrence. However, several limitations hamper the generalizability of these results. Notably, the study’s case–control design—with the control group, which underwent PVI alone, being retrospectively selected—and the nearly absent use of contact force-sensing ablation catheters, crucial in correctly localizing small gaps, may have influenced the outcomes. Akoum et al. [37] demonstrated that atrial fibrosis, present at baseline and unaltered by ablation-induced scarring, was directly associated with arrhythmia-free survival while reducing the residual fibrosis by ablation-scarring was independently associated with a lower recurrence rate of AF. Similar results were found in an entirely different cohort of patients [37].

Two independent randomized controlled trials (RCTs) have failed to show a significant improvement in AF outcomes when DE-CMR-guided substrate ablation is added to PVI in a population of both paroxysmal and persistent AF. The ALICIA trial did not demonstrate the additional benefit of targeting CMR-detected atrial fibrosis in addition to PVI in an unselected but relatively healthy population, with more than half of the subjects with paroxysmal AF and mildly dilated LA [38]. Notably, only about half of the patients exhibited fibrotic areas beyond the PV antrum that could be targeted, and when these areas were present, they were typically small and could be encompassed by solely encircling the pulmonary veins (PVs). To overcome this limitation, the DECAAF II RCT was designed to include only patients with advanced LA disease and persistent AF [39]. Although the relatively high atrial fibrosis burden with almost 50% of patients with a baseline atrial fibrosis of 20%, MRI-guided fibrosis-targeted ablation with PVI, compared with PVI alone, did not significantly improve atrial arrhythmia recurrence at 12–18 months.

## 7. Limitations of DE-CMR in Detecting Atrial Fibrosis

The DECAAF II trial’s failure to meet its primary endpoint has cast doubt on the clinical relevance of MRI-detected LA fibrosis, particularly since recent research has not demonstrated a clear link between the extent of fibrosis and AF recurrence [42]. The Utah method used for the quantification of diseased atrial regions and reported in the study by Marrouche et al. [25] and Akoum et al. [40] is based on the individual operator’s decision, who determines the intensity threshold to be chosen for LA-LGE detection [43]. To further increase reproducibility and decrease operator dependency, the image–intensity ratio (IIR) method was introduced. The mean intensity of the LA blood pool is used as the reference, with LA-LGE areas defined as regions where the signal intensity surpasses this reference by a predetermined factor. However, the three main studies aiming at assessing the IIR threshold for normal and dense scar tissue in patients with AF have not used a standard methodology, and derived cut-off values were mainly referring to heterogeneous cohorts of patients [44,45,46]. Indeed, no correlation has been found between IIR and Utah staging with electro-anatomical mapping, the latter correlating with freedom from arrhythmia recurrence in a population of ablation-naive patients with persistent AF [42]. Table 2 displays the LA CMR imaging post-processing protocol adopted in the most relevant studies evaluating the role of CMR in AF ablation strategy.

Notably, CMR acquisition (Supplementary Table S1) and post-processing (Table 2) protocols display several differences among studies, e.g., the time of T1 acquisition after contrast injection, the software for 3D atrial reconstruction models, or the LGE threshold for fibrotic tissue definition. At the same time, it remains uncertain whether DE-CMR effectively identifies atrial fibrosis as a true electrophysiological substrate. Indeed, a recent observational study demonstrated that endomysial fibrosis, rather than the overall connective tissue content, plays a key role in conduction disturbances in human AF. Moreover, this specific type of fibrosis has shown a good correlation with AF complexity, expressed as waves/cycle, as assessed by unipolar electro-anatomical mapping [47].

Another possible pitfall in DE-CMR guiding AF ablation is the identification of the LA wall border because the LA wall thickness is near the limit of image resolution and because of the proximity of hyperintense structures, i.e., the intima layer of the aortic wall, which could hamper the correct delineation of fibrosis areas [48]. To minimize endocardial and epicardial segmentation artifacts, atrial wall reconstruction with a mid-myocardial (50% thickness) layer has been introduced by ADAS software. Finally, yet importantly, fibrosis quantification methods and accessory thresholds have not undergone a large histological validation study in human AF hearts. While McGann et al. [26] showed a good correlation between left atrial tissue hyperenhancement on DE-MRI and human tissue biopsy findings in nine patients, the algorithm used to detect and quantify fibrosis in that study required an experienced observer to choose threshold levels, hampering the generalizability of these results.

## 8. DE-CMR in Guiding AF Substrate Ablation: Beyond the DECAAF II Results

The negative findings of the DECAAF II trial are consistent with those observed in previous studies that have targeted atrial fibrosis as defined by voltage mapping, showing comparable recurrence rates between patients with and without low voltage area ablation [9,49]. However, these results do not undermine the evidence from both DECAAF I and II and several other studies [31,36,37,41,50], which demonstrated that baseline atrial fibrosis identified through DE-MRI is predictive of ablation outcomes. Rather, DECAAF II leaves open the question of whether the absence of benefit stems from a lack of causal association between fibrosis and arrhythmia or from the limitations of conventional ablation techniques in effectively making durable fibrotic-targeted scarring tissue. This latter point seems crucial because the persistence of ablation-induced scarring at a distance from the procedure has been questioned by several observational studies [36,40], as residual fibrosis uncovered by ablation scarring is associated with recurrent arrhythmia [40]. The extensive pre-procedural and post-procedural DE-CMR imaging data in DECAAF II may provide valuable insights into this critical question.

## 9. Conclusions

We highlighted the growing body of evidence showing how MRI-derived fibrosis quantification may be predictive of procedural outcomes and arrhythmia recurrence while questioning its effective role in AF ablation strategy. DE-CMR can currently be considered a tool for identifying the ‘tip of the iceberg’, specifically in patients with extensive fibrosis and a very high risk of adverse events. However, it is not suitable for visualizing diffuse fibrosis, which poses a significant limitation given that atrial fibrosis is often a widespread process. Furthermore, the tissue characterization offered by CMR is not able to discriminate between reactive and replacement fibrosis, with different physio-pathological meanings and potentials to trigger and maintain AF. High-definition voltage mapping remains the mainstay in identifying substrates and guiding AF lesion formation, which is invaluable in the procedural phase. Variability in imaging protocols, along with a lack of standardization in fibrosis quantification and limited large-scale validation, represent important DE-CMR limitations that need to be addressed in future studies.

## Figures and Tables

**Table 1:** Most relevant studies evaluating the added value of DE-CMR in AF ablation strategy.

Study/Year	Methodology	Disease	Age; % Male; Patients	Comments	Results
Quinto et al./2020 [36]	Retrospective case–control	Paroxysmal AF (69%)	53 ± 9; 71%; 35 subjects	Shorter procedures and better clinical outcomes in repeated AF ablation procedures when DE-CMR is used in substrate characterization of anatomical veno-atrial gaps.	Positive
Bisbal et al./2020 (ALICIA) [38]	RCT	Paroxysmal AF (58%)	59; 72.4%; 76 subjects	CMR-guided substrate ablation did not improve the recurrence rate at 1 year of follow-up in the AF patient population undergoing first or repeat ablation procedures.	Negative
Marrouche et al./2022 (DECAAF II) [39]	RCT	Persistent AF (100%)	62.2; 79%; 421 subjects	CMR-guided fibrosis-targeted ablation with PVI did not significantly improve atrial arrhythmia recurrence at follow-up compared with PVI alone in persistent AF subjects.	Negative
Akoum et al./2015 [37]	Retrospective observational	Paroxysmal AF (53%)	66 ± 11; 64.5%; 172 subjects	Procedural outcomes are better anticipated by baseline atrial fibrosis and ablation-induced substrate alteration evaluated by CMR.	Positive
Akoum et al. /2015 [40]	Prospective observational	Paroxysmal AF (65%)	65.6%; 177 subjects	Baseline and residual atrial fibrosis uncovered by ablation scarring are associated with recurrent arrhythmia.	Positive
Marrouche et al./2014 [25]	Prospective observational	Paroxysmal AF (65%)	64.6%, 260 subjects	Baseline atrial fibrosis estimated by DE-CMR is associated with arrhythmia recurrence.	Positive
Ferrò et al./2023 [31]	Retrospective observational	Paroxysmal AF (66%)	57 ± 11, 72%, 166 subjects	Advanced atrial cardiomyopathy detected by DE-CMR is independently associated with the recurrence of arrhythmia, particularly atypical atrial flutter, after AF ablation.	Positive
Chelu et al./2018 [41]	Retrospective observational	Paroxysmal AF (40%)	65 ± 12, 60%, 308 subjects	The extent of left atrial fibrosis predicts the 5-year outcome of atrial fibrillation ablation.	Positive

AF: atrial fibrillation; CMR: cardiac magnetic resonance; DE: delayed enhancement; PVI: pulmonary vein isolation; RCT: randomized controlled trial.

**Table 2:** Atrial CMR imaging post-processing protocol adopted in the most relevant studies evaluating MRI-guided AF ablation.

First Author/Year/Design of the Study	CMR Post-Processing Protocol
Marrouche/2014/The DECAAF study: a multicenter, prospective, observational cohort study [25]	Following data acquisition, CMR data were evaluated and processed using the Coreview image processing software (MARREK, Salt Lake City, UT, USA) for the processing of the LA wall segmentation, fibrosis identification, and export of 3D models.
Marrouche/2022/The DECAAF II study: RCT [39]	Patients received a DE-MRI within 30 days before the ablation procedure, using the Merisight protocol from MARREK Inc., to quantify left atrial fibrosis in each case. Reviewers who evaluated the quality of the MRIs were blinded to the patients’ randomized treatment groups. MARREK Inc provided support for image segmentation, processing, and the quantification of left atrial fibrosis. After the ablation, follow-up DE-MRIs were conducted between 90 and 180 days later to assess the formation of scar tissue related to the procedure. The reviewers classified the amount of fibrosis on a five-level scale.
Bisbal/2020/The ALICIA study: RCT [38]	Image post-processing of DE-CMR images was conducted using the dedicated ADAS software to obtain a 3D reconstruction of the LA incorporating fibrosis information. Experienced operators manually outlined the LA mid-wall contours in the axial plane to obtain the initial 3D model; further shell deformations were performed, if necessary. Fibrosis identification was based on voxel signal intensity and the mean signal intensity of the LA blood pool was automatically calculated; then, the image intensity ratio value for each voxel was calculated (voxel signal intensity/mean pixel intensity of blood pool) and projected to the 3D model to obtain the final fibrosis map. The thresholds for fibrosis visualization were 1.20 (native fibrosis) and 1.32 (dense scar).
Akoum/2015/monocentric, retrospective observational study [37]	Left atrial wall volumes were manually segmented from the LGE-MRI by expert observers using the Coreview image processing software (Marrek Inc.); the resulting LA wall segmentation included the 3D extent of both the LA wall and the antral regions of the PVs. Fibrosis regions in the LGE-MRI were identified based on an intensity threshold established through expert evaluation. A volume-rendering tool in Corview enabled operators to visualize enhancement distribution in 3D; a custom transfer function was employed to define enhancement gradations while suppressing blood and normal tissue. Fibrosis and scarring were reported as a percentage of the atrial wall; patients were categorized into four stages (Utah stages) based on their baseline fibrosis.
Quinto/2020/case–control study [36]	All DE-CMR images were segmented by experienced observers with ADAS3D software (ADAS3D Medical, Barcelona, Catalonia, Spain). The atrial wall was manually traced in axial slices, and the blood pool was automatically calculated. Color-coded pixel signal intensity maps were projected to the 3D shell of the atrium. Pixel signal intensity maps were normalized to the mean intensity of the blood pool signal, and the resulting value was plotted. Previously validated fibrosis thresholds were used, with an IIR > 1.32 determining dense scar areas and an IIR between 1.2 and 1.32 determining interstitial fibrosis. The resulting processed 3D model of the LA DE-CMR was integrated with the electro-anatomical map of the navigation system (CARTO 3, Biosense Webster, Diamond Bar, CA, USA) to guide the ablation procedure.
Ferrò/2023/retrospective observational study [31]	For the reconstruction of LA and the right atrium, the atrial walls were manually traced. LGE was defined using an IIR > 1.2, indicative of fibrotic tissue. LA was automatically divided into seven standardized regions.
Chelu/2018/retrospective observational [41]	The relative extent of fibrosis was quantified within the LA wall with a threshold-based algorithm. The algorithm automatically determines a threshold intensity likely to correspond to the enhanced or fibrotic voxels of the left atrium by estimating the mean and standard deviation of “normal” tissue. “Normal” tissue is defined as the lower region of the pixel intensity histogram, ranging from 2% to 40% of the maximum intensity within the region of interest (e.g., the left atrial wall). The threshold for enhanced/fibrotic tissue is then set at two to four standard deviations above the mean of the “normal” tissue, covering approximately 95% to 99.994% of a Gaussian distribution.

3D: three-dimensional; AF: atrial fibrillation; CMR: cardiac magnetic resonance; DE: delayed enhancement; IIR: image–intensity ratio; LA: left atrium; MRI: magnetic resonance imaging; PV: pulmonary vein; RCT: randomized clinical trial.

## Data Availability

Not applicable.

## Supplementary Materials

The following supporting information can be downloaded at: https://www.mdpi.com/article/10.3390/jcdd12040114/s1, Table S1: Atrial CMR imaging acquisition protocol adopted in the most important studies evaluating MRI-guided AF ablation.
