# Supplementary material for: The Role of Cardiac Magnetic Resonance in Characterizing Atrial Cardiomyopathy and Guiding Substrate Ablation in Atrial Fibrillation: A Narrative Review
Source: J Cardiovasc Dev Dis. 2025 Mar 25;12(4):114. doi: 10.3390/jcdd12040114 (PMC12027483; doi:10.3390/jcdd12040114)
Supplement: Supplementary file 1 [file jcdd-12-00114-s001.zip › jcdd-3538792-supplementary.pdf]

Supplementary table 1. Atrial CMR imaging acquisition protocol adopted in the most important studies evaluating MRI-guided AF ablation.

3D: three dimensional; AF: atrial fibrillation; CMR: cardiac magnetic resonance; DE: delayed enhancement; FOV: field of view; GRAPPA: Generalized Autocalibrating Partially Parallel Acquisition; IR: inversion recovery; IV: intravenous; LA: left atrium; MRI: magnetic resonance imaging; NA: not available; PV: pulmonary vein; PVI: pulmonary vein isolation; RCT: randomized clinical trial; T: Tesla; TI: inversion time; TE: echo time; TR: repetition time.

First author / year / Design of the study	CMR acquisition protocol
Marrouche / 2014 / The DECAAF study: a multicenter, prospective, observational cohort study [25]	Pulse sequences were acquired to evaluate LA and PV anatomy and LA fibrosis. High resolution 3D DE-CMR images of the LA were acquired approximately 15 minutes after contrast injection using a 3D ECG-gated, respiratory-navigated, inversion-recovery prepared gradient echo pulse sequence. Inversion preparation was applied at every heartbeat, and fat saturation was performed immediately before data acquisition. Data acquisition was limited to 15% of the averaged cardiac cycle and was performed during left atrial diastole. The scan parameters for DE-MRI of the LA at a 3 T scanner were as follows: axial imaging volume with a FOV of 400 × 400 × 110 mm, voxel size of 1.25 × 1.25 × 2.5 mm, TR of 3.1 ms, TE of 1.4 ms, and a flip angle of 14°. For DE-MRI of the LA at a 1.5 T scanner, the parameters were: FOV of 360 × 360 × 100 mm, voxel size of 1.25 × 1.25 × 2.5 mm, TR/TE

	of 5.2/2.4 ms, and a flip angle of 20°. Depending on patient respiration, the typical scan time for DE-MRI was 6–12 minutes at 1.5 T and 5–9 minutes at 3T. Each participating center followed its standard clinical protocol for contrast injection in cardiac MRI.
Marrouche / 2022 / The DECAAF II study: RCT [39]	NA
Bisbal / 2020 / The ALICIA study: RCT [38]	One to 30 days before the scheduled procedure, a CMR was performed using a 3 T scanner with a dedicated 32-channel cardiac coil or a 1.5 T scanner with a 16-channel cardiac coil. DE-CMR scans were acquired 20-40 minutes after contrast injection using a free-breathing 3D navigator and ECG-gated IR-gradient-echo sequence applied in the axial orientation (voxel size: 1.25×1.25×2.5 mm). Other parameters: TR/TE 2.3 to 3/1.4 to 2.1 ms; flip angle, 11 to 19°, bandwidth, 460 to 255 Hz/pixel; inversion time 280 to 360 ms and parallel imaging with the GRAPPA technique, with reference lines of R=2 and 72. An inversion time scout sequence was employed to eliminate the left ventricular myocardial signal and determine optimal inversion time. The average scan duration for a DE-CMR sequence was around 15 minutes, influenced by heart rate and breathing patterns. Electrical cardioversion was performed, if required, to maintain sinus rhythm during image acquisition.

Akoum / 2015 / monocentric, retrospective observational study [37]	All patients underwent LGE-MRI prior to catheter ablation and three months following ablation. All scans were performed using a 3-T Verio clinical scanner. High-resolution LGE images of the LA were acquired about 15 minutes following contrast injection using a 3D, IR-prepared, respiration-navigated, ECG-gated, gradient echo pulse sequence. Typical acquisition parameters were free breathing using respiratory navigation, a transverse imaging volume with voxel size = 1.25 × 1.25 × 2.5 mm (reconstructed to 0.625 × 0.625 × 1.25 mm), TR/TE = 3.1/1.4 ms, flip angle = 14°, TI = 280–330 ms, and GRAPPA with reduction factor of 2. Inversion pulse was applied every heartbeat, and fat saturation was applied immediately before data acquisition. Data acquisition was limited to 15% of cardiac cycle during the diastolic phase of the atrial cardiac cycle. The TE of the scan (1.4 ms) was chosen such that fat and water were almost out of phase and the signal intensity of partial volume fat-tissue voxels was reduced allowing improved delineation of the atrial wall boundaries. The TI value for the LGE-MRI scan was identified using a TI scout scan. Typical scan time for the LGE-MRI study was four to nine minutes depending on subject respiration.
Quinto / 2020 / case-control study [36]	Patients undergoing a DE-CMR-guided PVI redo procedure received a DE-CMR prior to the ablation procedure to identify potential PV gaps. Electrical cardioversion was performed, if required, to maintain sinus rhythm during image acquisition.

	A 3 T scanner using a 32-channel cardiac coil was used for all patients. After 20 min of IV contrast administration, a free-breathing 3D navigator and ECG-gated IR gradient-echo sequence were applied in axial projection (acquired voxel size: 1.25 x 1.25 x 2.5 mm). Other typical sequence parameters included: repetition time/echo time, 2.3/1.4 ms; flip angle, 11; bandwidth, 460 Hz/pixel; TI 280–380 ms; and parallel imaging with GRAPPA technique, with reference lines of R= 2 and 72. A TI scout sequence was used to nullify the left ventricular myocardial signal and determine optimal TI. The typical scan time for a DE-CMR sequence was approximately 15 minutes, depending on the patient's heart rate and breathing patterns.
Ferrò / 2023 / retrospective observational study [31]	Inversion recovery-prepared T1 weighted gradient-echo sequences were acquired in axial orientation 20 minutes after contrast administration. The sequence parameters were as follows: repetition time of 2.3 ms, echo time of 1.4 ms, flip angle 11°, bandwidth of 460 Hz/pixel, inversion time of 280-380 ms and acquired voxel size of 1.25 x 1.25 x 2.5 mm.
Chelu / 2018 / retrospective observational [49]	Scans were performed 15 minutes after the injection of a contrast agent using a 3D inversion recovery-prepared, respiration-navigated, ECG-gated gradient echo pulse sequence. The acquisition parameters included free breathing with navigator gating, a transverse imaging volume with a voxel size of 1.25×1.25×2.5 mm (reconstructed to

	0.625×0.625×1.25 mm), an inversion time of 270 to 320 ms, and GRAPPA with a reduction factor of R=2.
